# Giant renal angiomyolipoma unmasks underlying lymphangioleiomyomatosis: a case report and literature review

**DOI:** 10.3389/fmed.2025.1610464

**Published:** 2025-08-01

**Authors:** Xuan Zhou, Qiaoling Chen, Zhiyong Xiong, Miao Wang, Xiaoping Zhang

**Affiliations:** ^1^Department of Urology, Union Hospital, Tongji Medical College, Huazhong University of Science and Technology, Wuhan, Hubei, China; ^2^Institute of Urology, Union Hospital, Tongji Medical College, Huazhong University of Science and Technology, Wuhan, Hebei, China; ^3^Department of Pulmonary and Critical Care Medicine, Peking Union Medical College Hospital, Beijing, China; ^4^Chinese Academy of Medical Sciences, Peking Union Medical College, Beijing, China; ^5^Shenzhen Huazhong University of Science and Technology Research Institute, Shenzhen, China

**Keywords:** lymphangioleiomyomatosis, renal angiomyolipoma, diagnosis and treatment, mTOR inhibitors, case report

## Abstract

Lymphangioleiomyomatosis (LAM) is an uncommon systemic disease that primarily affects women during their reproductive years and is often linked with renal angiomyolipoma (AML). In this report, we describe a case involving a 30-year-old female patient who presented with a large AML in her right kidney. Imaging revealed diffuse pulmonary cysts, raising clinical suspicion of LAM. The procedure was initially planned as a minimally invasive partial nephrectomy performed with robotic assistance but was converted to open radical nephrectomy due to excessive intraoperative bleeding. Histopathological analysis confirmed epithelioid AML, and genetic testing revealed a somatic TSC2 mutation, further supporting the diagnosis of sporadic LAM. This case highlights the need to consider LAM in young women with renal AML and emphasizes the value of multidisciplinary management in addressing such complex clinical scenarios.

## 1 Introduction

Lymphangioleiomyomatosis (LAM), also known as lymphangiomyomatosis, is a slow progressive rare neoplastic disease that predominantly affects women (1). LAM characterized by the uncontrolled proliferation of smooth muscle cells in the lungs, pulmonary vessels, lymphatic vessels, and pleura, leading to structural deformation of lung tissue, cystic emphysema, and progressive deterioration of lung function. LAM can manifest either independently (sporadic LAM) or in conjunction with tuberous sclerosis complex (TSC). Renal angiomyolipoma (AML), also known as renal hamartoma, is a significant manifestation of LAM. This article reviews the clinical data and treatment course of a case of sporadic LAM (S-LAM) with an uncommon giant renal AML, and addresses the diagnostic and therapeutic challenges associated with complex AML. By integrating relevant literature and current treatment guidelines, it offers insights for future clinical practice.

## 2 Case presentation and methods

### 2.1 Clinical data

**Figure 1:**
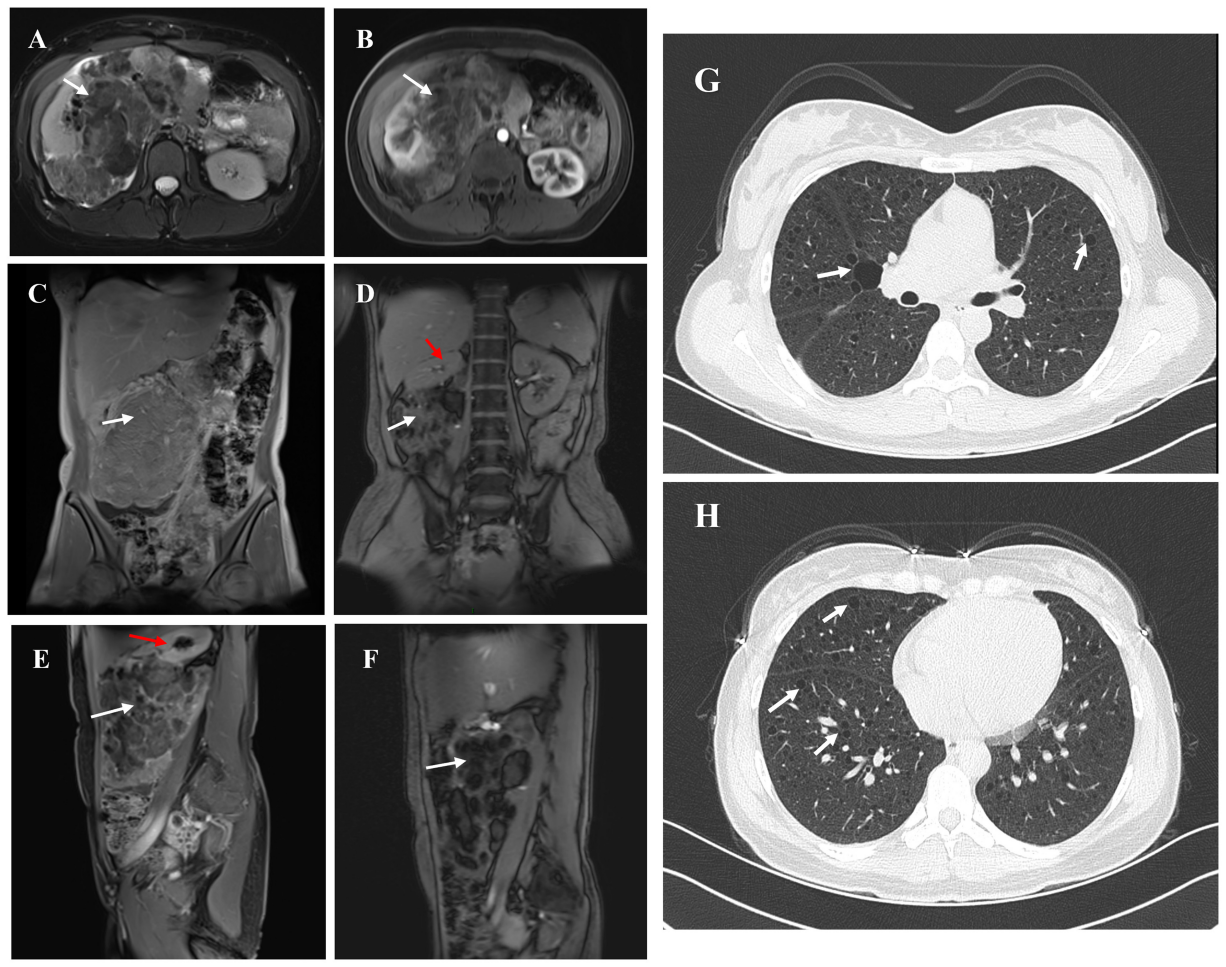
The figure summarizes the findings from imaging examinations of the primary tumor lesion. **(A, B)** Non-contrast AND Arterial phase contrast-enhanced MRI. White arrow indicates right renal angiomyolipoma and red indicates the remaining normal tissue of the right kidney. **(C–F)** Coronal/Sagittal MRI showing a giant right renal angiomyolipoma. **(G, H)** Chest CT. White arrow indicates the Thin-walled cystic lesions in both lungs.

A 30-year-old female patient was admitted to the hospital after a physical examination revealed a right renal mass 1 month prior. She had no symptoms such as chest tightness, cough, dyspnea, abdominal distension, abdominal pain, or hematuria. She was unmarried, had no history of smoking or alcohol consumption, and had no significant past medical or family history. Physical examination showed no obvious abnormalities. Routine blood and urine tests, biochemical tests, and coagulation tests all showed no significant abnormalities. Enhanced MRI of the retroperitoneum and pelvis revealed a large mass in the right abdomen, pelvis, and retroperitoneum, measuring approximately 16.3 cm × 16.3 cm × 8.4 cm, with heterogeneous slightly long T1 and T2 signals (Figures 1A–F). Diffusion-weighted imaging (DWI) showed significant diffusion restriction, and the mass exhibited heterogeneous enhancement on contrast scans. The mass crossed the midline, partially encased the right kidney, superior mesenteric vein, and right renal vessels, and caused compression of surrounding structures, including upward displacement of the right kidney, leftward displacement of the pancreatic head and abdominal intestines, and a small amount of ascites. A nodular short T1 and T2 signal was observed on the anterior upper wall of the uterus, measuring approximately 13 mm × 10 mm × 13 mm, with no significant enhancement on contrast scans, suggesting a possible submucosal uterine fibroid. Ultrasound of the inferior vena cava showed unclear visualization of the lower segment, possibly due to compression. Color Doppler ultrasound of the superior mesenteric vein and right renal vein showed no obvious tumor thrombus. Chest CT revealed multiple thin-walled cystic lucencies of varying sizes in both lungs, with the largest located in the right lung, measuring ~22 mm in length, suggestive of lymphangioleiomyomatosis. Scattered micronodules, ~2–3 mm in size, were also observed in both lungs. Multiple lymph nodes were visible in the mediastinum, with a cross-sectional size of ~9 × 5 mm. No significant lymphadenopathy was observed in the hilar regions, and the trachea and bronchi were patent (Figures 1G, H). Pulmonary function tests were within normal limits, with a decreased maximum expiratory flow at 25% of forced vital capacity. Brain MRI showed no significant abnormalities.

### 2.2 Surgical procedure

**Figure 2:**
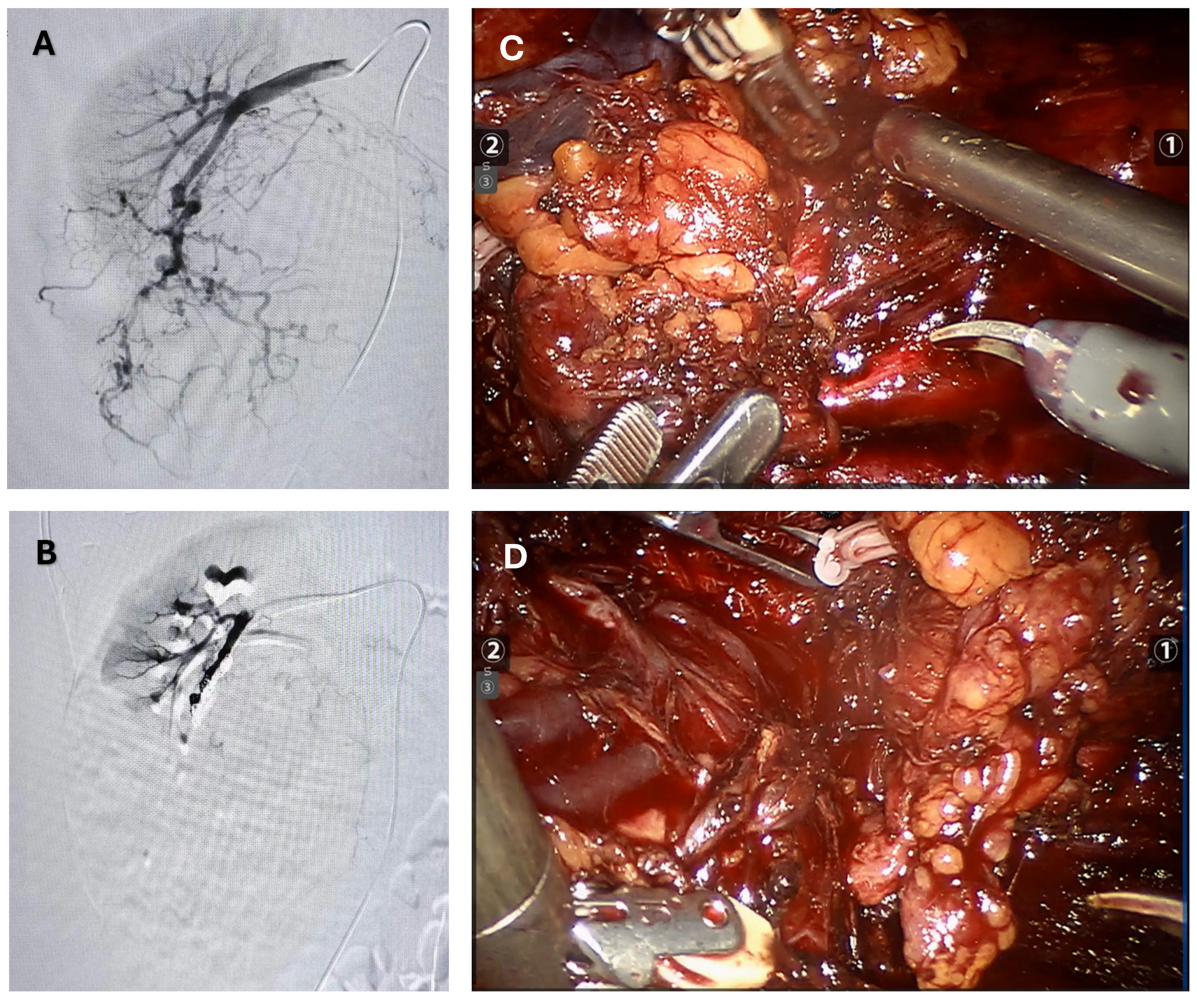
The figure summarizes the preoperative and intraoperative findings. **(A)** Pre-embolization angiography: Extensive arterial vessels within the right renal angiomyolipoma. **(B)** Post-embolization angiography: No visualization of arterial vessels within the right renal angiomyolipoma. **(C, D)** During surgery, a giant renal angiomyolipoma was identified. The lesion was encapsulated and showed a mixed consistency, featuring significant fat content along with regions of soft, vascular tissue and localized hemorrhage.

The patient was preoperatively diagnosed with a right renal tumor, with malignancy not excluded. MRI and ultrasound indicated a large tumor with complex surrounding structures and a high risk of rupture and hemorrhage. Considering the surgical risks, the patient's age, and her wishes, a robot-assisted laparoscopic partial nephrectomy was planned. To reduce intraoperative bleeding, the patient underwent selective arterial embolization of the right renal tumor 1 day before surgery. Angiography showed thickening and disorganization of the lower pole branch vessels of the right renal artery, with abnormal staining during the parenchymal phase (Figure 2A). Embolization was performed by injecting 300–500 μm and 500–700 μm gelatin sponge particles into the target vessels, followed by placement of two 6 mm × 14 mm and one 8 mm × 14 mm coils. Post-embolization angiography showed successful occlusion of the tumor vessels (Figure 2B). The patient was transferred back to the ward with a surgical drain.

The next day, the patient underwent laparoscopic partial nephrectomy performed with robotic assistance. Following induction of anesthesia, the patient was positioned in the left lateral decubitus posture, and trocars were introduced in sequence. The perirenal adipose tissue was carefully separated to reveal the renal artery and vein. After releasing perirenal adhesions, the renal lesion was exposed. The tumor was sizable, involving the renal vessels and overlaying the vena cava (Figures 2C, D). Its soft texture and marked vascularity caused immediate bleeding on contact, making bleeding control difficult. Due to tight adhesions and abundant feeding vessels, dissection of the renal hilum and ureter resulted in significant bleeding, obscuring the surgical field and making kidney preservation difficult. After discussing with the family members of the patience, a radical right nephrectomy was determined to perform. Hem-o-lock clips were used to ligate the renal artery and vein, which were then transected. Following dissection along the renal capsule, the ureter was ligated with Hem-o-lok clips and divided. The right kidney was completely excised and submitted for pathological examination. After trocar removal, the incision was closed in layers, and a drainage tube was left in situ.

### 2.3 Postoperative management

**Figure 3:**
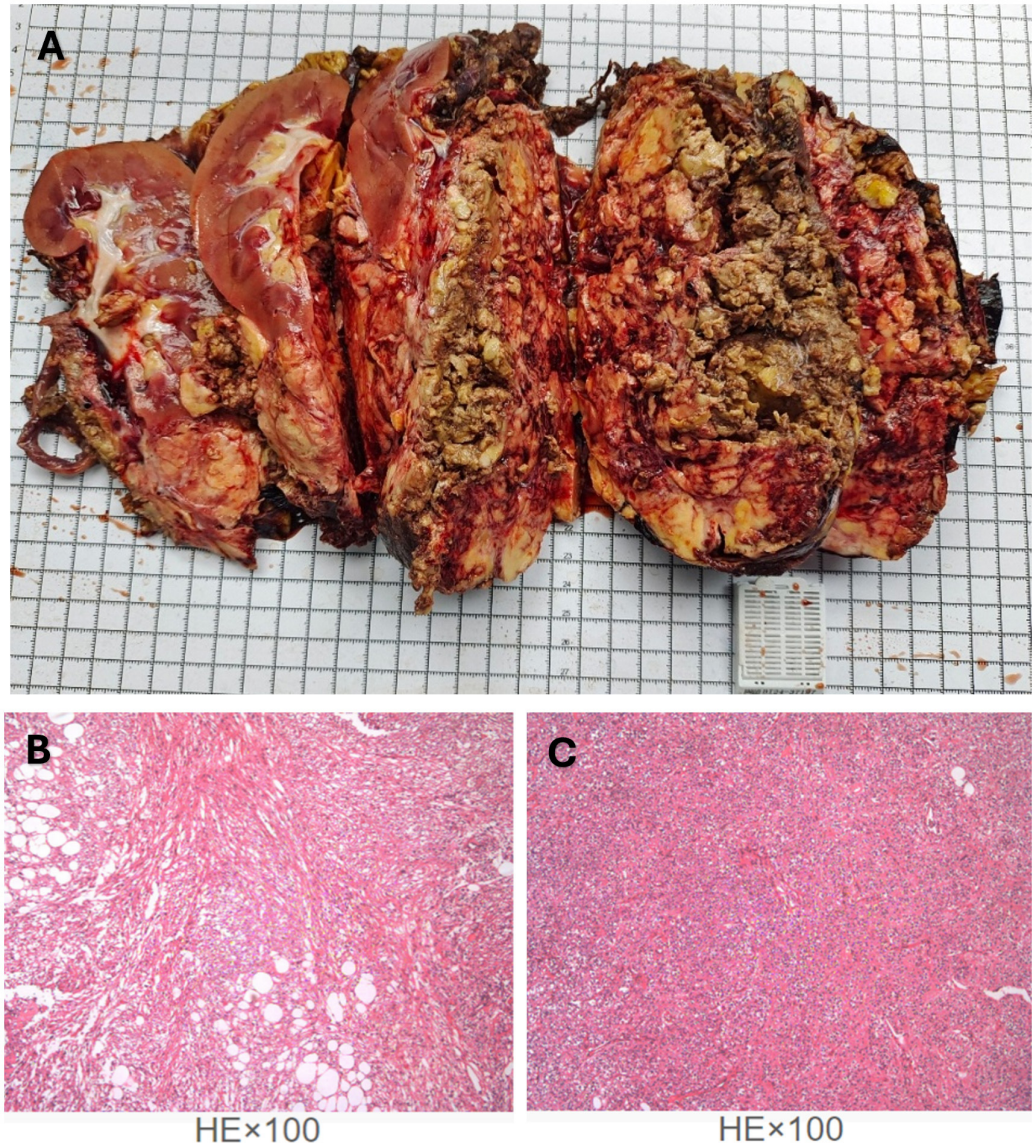
The legend illustrates the gross appearance and pathological findings of the specimen following tumor resection. **(A)** Postoperative pathological specimen of the right renal angiomyolipoma. **(B, C)** Hematoxylin–eosin (HE) staining (magnification × 100) showed the structures of epithelioid angiomyolipoma morphology.

The patient recovered well, started a semi-liquid diet on the third postoperative day, had the drainage tube removed on the seventh day, and was discharged on the ninth day. The postoperative pathological report described a nodular mass measuring 20 cm × 13 cm × 12 cm, with a grayish-white, yellowish, and reddish cut surface, soft texture, and partial encapsulation (Figure 3A). Attached to one side was renal tissue measuring 12 cm × 6 cm × 4 cm, with smooth renal pelvis and calyces mucosa. The attached ureter measured 13 cm × 0.5 cm. The pathological diagnosis was (right renal mass and retroperitoneal mass) angiomyolipoma, with some areas showing epithelioid angiomyolipoma morphology (Figures 3B, C). The surgical margins were negative. Immunohistochemical staining showed Smooth Muscle Actin (SMA) (+), Desmin (partial+), S100 (partial+), Human Melanoma Black 45 (HMB45) (focal+), Melan A (partial+), Epithelial Membrane Antigen (EMA) (–), CD34(–), SOX10(–), and Ki67 (LI: 2%).

Pan-cancer gene next-generation high-throughput sequencing (NGS) testing: based on probe capture technology and Illumina NGS, the Tianjin Jiankang Huamei solid tumor gene detection 785-gene NGS kit was used. The results showed two potentially clinically significant somatic mutations in the TSC2 gene (Class II variants): Intron 8 splice donor mutation c.774+1G>C and Exon 15 p.Arg505 nonsense mutation. Peripheral blood samples were tested using multiple NGS panels, and no TSC1/2 germline mutations were detected.

Five months after surgery, the patient underwent a follow-up renal ultrasound, which showed no significant abnormalities in the left kidney and a generally filled bladder with no obvious abnormalities. The left ureter was not dilated. Renal function tests showed a slightly decreased glomerular filtration rate of 78.57 ml/(min·1.73^2^), with other parameters within normal limits. Additionally, the Vascular Endothelial Growth Factor-D (VEGF-D) level was measured at 520 pg/mL. The patient reported no significant discomfort.

## 3 Discussion

LAM is an uncommon systemic disorder that mainly affects women and primarily targets the lungs (1). Extrapulmonary manifestations include renal AML, retroperitoneal AML, or cystic-solid masses. A multinational study found that the prevalence of LAM in women is 3.4–7.8 per million (2). LAM may present as a sporadic condition or be associated with TSC. Unlike TSC patients with germline mutations in TSC1 or TSC2 (where the mutation is inherited in a mendelian manner or occurs early in embryogenesis, affecting all cells), sporadic LAM patients only have somatic mutations in the TSC genes (limited to the affected tissues). Therefore, sporadic LAM is not hereditary, which is consistent with the genetic testing results in our case.

Of course, routine TSC gene testing is not necessary for the diagnosis of LAM. The sensitivity of genetic testing for suspected TSC-LAM is not 100%, so a negative result does not exclude the disease. Approximately 10–15% of patients clinically diagnosed with TSC do not have detectable TSC1/2 germline mutations using conventional exon sequencing methods (3), possibly due to functional germline mutations in intronic regions not covered by NGS probes or mosaic mutations (4, 5).

Renal angiomyolipoma (AML) is a noncancerous tumor consisting of blood vessels, smooth muscle, and fat. Clinically, it is classified as either sporadic or linked to TSC. Approximately 30–40% of sporadic LAM patients develop AML, while over 90% of women with TSC-LAM may develop AML (2). Small lesions may be asymptomatic, but lesions larger than 4 cm (especially those containing aneurysms) may cause symptoms such as abdominal pain and a palpable mass (6, 7). When AML is large, bilateral, or multiple, TSC should be ruled out first. In addition to the fact that TSC often involves multiple systems, the growth rate of AML may also differ. Research conducted by Seyam et al. reported that sporadic renal AML exhibits an average growth rate of 0.19 cm per year, whereas TSC-related AML increases in size at approximately 1.25 cm annually (8).

In this case, although the presence of a giant AML was rare and initially raised suspicion of malignancy or TSC, the absence of other typical clinical features of TSC, such as shagreen patches, subependymal nodules, hypopigmented macules, or seizures (Table 1), did not support a clinical diagnosis of TSC (9). Notably, the 2012 International TSC Conference Diagnostic Consensus clearly states that the presence of renal AML and LAM alone is not sufficient for a diagnosis of TSC (10). Based on the patient's postoperative pathology and genetic testing results, after thorough discussion with the pathology and respiratory departments, the final diagnosis was sporadic LAM with AML, rather than TSC-LAM.

**Table 1:** Diagnostic criteria for tuberous sclerosis complex (TSC).

**Disease**	**Diagnostic criteria**	**Features**
Tuberous Sclerosis Complex (TSC)	Major features	1. Hypomelanotic macules (≥3, minimum diameter 5 mm)
		2. Angiofibromas (≥3) or forehead fibrous plaque
		3. Ungual fibromas (≥2)
		4. Shagreen patch
		5. Multiple retinal angiomyolipomas (AMLs)
		6. Cortical dysplasia (including cortical tubers and radial migration lines)
		7. Subependymal nodules
		8. Subependymal giant cell astrocytoma
		9. Cardiac rhabdomyoma
		10. Lymphangioleiomyomatosis (LAM)
		11. Renal angiomyolipomas (RAML) (≥2)
	Minor features	a. “Confetti” skin lesions
		b. Dental enamel pits (>3)
		c. Oral fibromas (≥2)
		d. Retinal pigment spots
		e. Non-RAML lesions
		f. Multiple renal cysts
A diagnosis of TSC is confirmed by the presence of either 2 major		
features or 1 major feature plus at least 2 minor features.		

This table summarizes the current clinical diagnostic symptoms and criteria for tuberous sclerosis.

When characteristic cystic changes of LAM are found on chest CT in young women, a clinical diagnosis of LAM can be made in combination with other features such as TSC, AML, and chylous effusions (Table 2). However, cystic lung diseases like emphysema, Langerhans cell histiocytosis, and Birt-Hogg-Dubé syndrome can sometimes exhibit symptoms that resemble those of LAM (11). Since High-Resolution Computed Tomography (HRCT) is not routinely performed, many LAM patients are misdiagnosed or undiagnosed, delaying the treatment. AML is the most frequent extrapulmonary feature of LAM, found in as many as 50% of cases (12), and may lead to symptoms such as pain, abdominal distension, and bleeding, particularly when the lesion exceeds 4 cm in size (6, 7). There is currently no specific data or research indicating the proportion of LAM patients presenting with initial symptoms. However, in some cases with severe disease or poor prognosis, extrapulmonary lesions often draw attention leading to diagnosis. In one rare case of male LAM, AML was diagnosed 10 years after surgery, with no renal or other clinical symptoms at the time of surgery (13).

**Table 2:** Algorithm for the diagnosis of lymphangioleiomyomatosis (LAM).

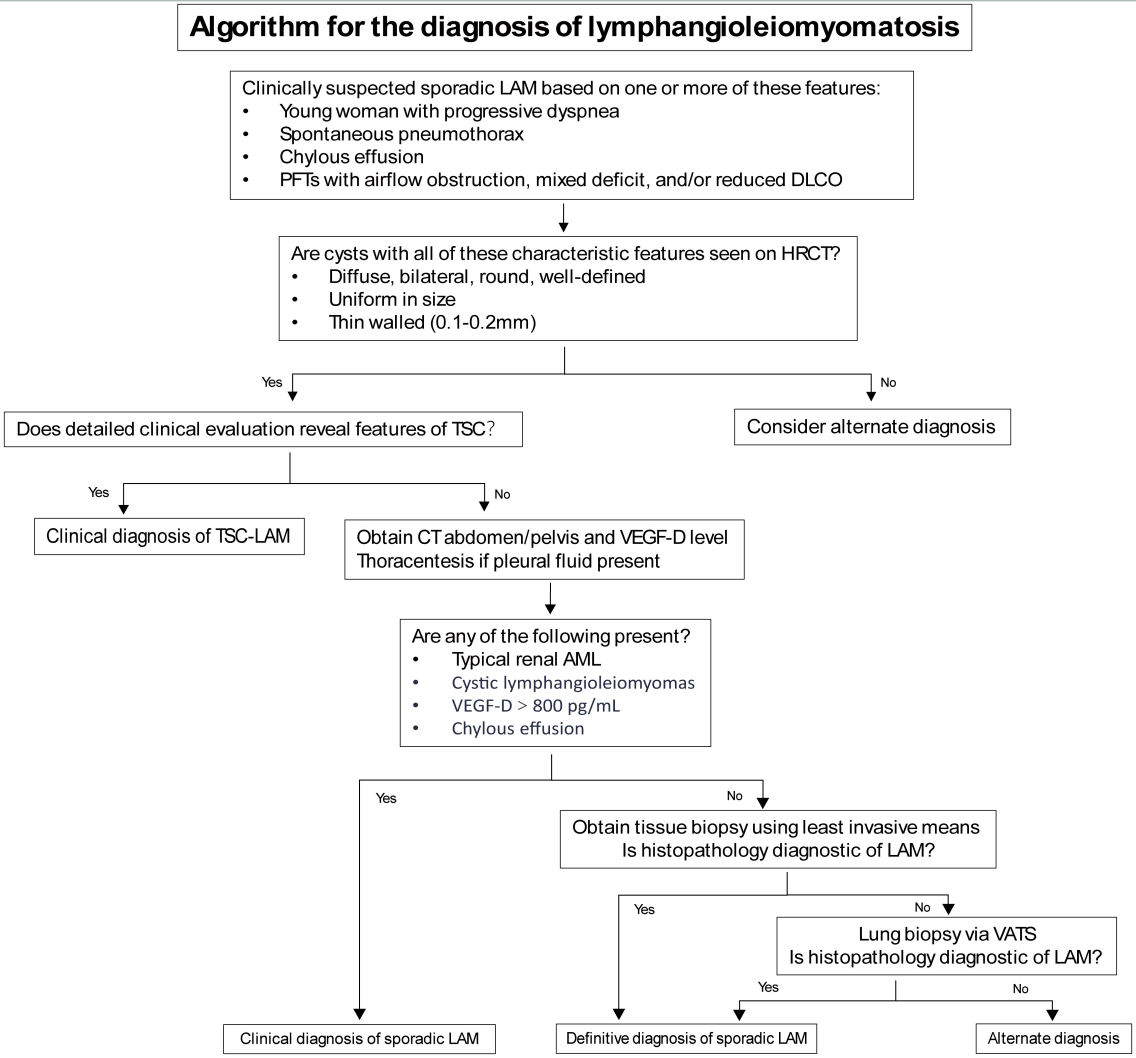

The algorithm for the diagnosis of LAM. This table is reproduced with permission from UpToDate and is intended for academic exchange and educational purposes only. PFTs, pulmonary function tests; DLCO, diffusing capacity carbon monoxide; HRCT, high resolution computed tomography; VEGF-D, vascular endothelial growth factor-D; VATS, video-assisted thoracoscopic surgery.

Pathological biopsy is the gold standard for LAM diagnosis, with immunohistochemical features showing positivity for α-smooth muscle actin (α-SMA), melanoma-associated antigen HMB45, and, as in this case, desmin. Estrogen and progesterone receptors in the lesion tissue are often positive. In current clinical practice, due to the risks and invasiveness, routine lung biopsy is not performed for pulmonary lesions. However, for AML requiring surgical intervention, postoperative pathology and immunohistochemistry can provide valuable diagnostic information. When necessary, genetic testing of the tumor can also help confirm the clinical diagnosis.

The treatment of LAM includes general measures and symptomatic treatment of pulmonary and extrapulmonary lesions (Table 3). General measures include oxygen therapy, bronchodilators, respiratory rehabilitation, and complication management. With advances in basic research, mTOR inhibitors such as sirolimus have been approved by the Food and Drug Administration (FDA), European Medicines Agency (EMA), and regulatory agencies in Japan, South Korea, Brazil, Russia, and Uruguay for the treatment of LAM. However, in China, sirolimus oral formulations are only approved for anti-rejection in kidney transplantation, and its use in LAM is off-label, requiring adherence to expert consensus guidelines (14). Sirolimus can slow the progression of respiratory function decline, but there is currently no cure for LAM.

**Table 3:** Lymphangioleiomyomatosis treatment measures.

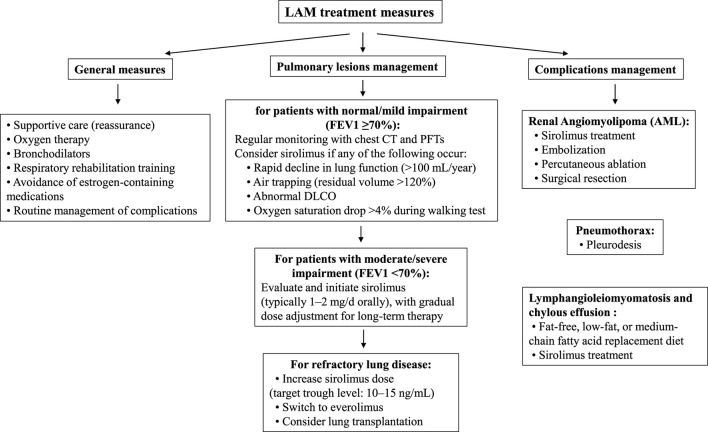

This table summarizes the key aspects and treatment strategies commonly used in the management of LAM.

Primary Pulmonary Lesions—When the forced expiratory volume in one second (FEV1) is at least 70% of the predicted value and there are no symptoms, no medication is needed, and regular monitoring of chest CT and pulmonary function (every 12–24 months) is sufficient. If FEV1 is <70% of the predicted value, or if there is rapid decline in pulmonary function (>100 mL/year), air trapping (residual volume >120%), abnormal diffusing capacity of the lungs for carbon monoxide (DLCO), or a drop in oxygen saturation >4% during walking, chronic administration of sirolimus could be evaluated as a therapeutic option. For severe pulmonary lesions, surgical lung transplantation may also be considered.

Renal Angiomyolipoma (AML)—As a common symptom in sporadic LAM patients, some lesions are unilateral, single, and asymptomatic, usually requiring no treatment. However, if there is an aneurysm >5 mm in diameter or, as in this case, a giant AML that may continue to grow, early intervention is necessary. Current treatment options include surgical resection, arterial embolization, percutaneous ablation, and oral mTOR inhibitors.

1. Surgical resection: nephron-sparing surgery (NSS), which aims to preserve as much normal renal tissue as possible, is now the most common surgical option for AML. For some AML cases with less ideal locations, robot-assisted surgery may offer advantages in terms of visualization and operative space.
2. Arterial embolization: the application of selective arterial embolization (SAE), particularly in hemodynamically unstable patients experiencing tumor rupture and bleeding, is frequently favored for its minimally invasive nature. However, the recurrence rate of AML after SAE alone may be higher than after NSS, with literature reporting rates ranging from 11% to 40% (15).
3. Percutaneous Ablation: this is usually limited to small, asymptomatic tumors with a clear preoperative diagnosis.
4. Pharmacological Treatment: for patients who cannot undergo surgery, embolization, or ablation, oral mTOR inhibitors may be an option. Sirolimus, the first mTOR inhibitor, not only inhibits AML growth but can also significantly reduce tumor volume in some cases (16).

Pneumothorax—For the first occurrence of pneumothorax, pleurodesis is recommended to reduce the risk of recurrence. Although pleurodesis may increase the difficulty of lung dissection in future lung transplant surgeries, it is not a contraindication.

Chylothorax and Lymphangioleiomyoma—For chylothorax, a fat-free or low-fat diet or substitution with medium-chain triglycerides is recommended. Sirolimus may also be considered for the treatment of chylothorax and lymphangioleiomyoma.

For patients with sporadic LAM and AML, timely diagnosis and early intervention in pulmonary and extrapulmonary disease progression are crucial for prognosis. LAM is clinically rare and involves multiple systems, placing high demands on the knowledge base of frontline physicians. Even for surgical specialists, when facing a relatively clear diagnosis of AML, it is essential to thoroughly integrate clinical symptoms, imaging studies, and laboratory tests to rule out LAM, TSC, and other possible diseases.

The treatment of complex AML has shown promising directions with the exploration of mTOR inhibitors. In a cohort of 61 renal angiomyolipoma patients enrolled in a phase II clinical trial, sirolimus administration for 12 months was associated with a 53% decline in tumor volume, though discontinuation led to volume rebound (16). Consistent outcomes were replicated in two independent studies (17, 18). A separate randomized controlled trial investigated everolimus (10 mg/day) in this population. Post 6-month follow-up, 55% of participants receiving everolimus attained ≥50% tumor reduction, with 80% demonstrating ≥30% volumetric regression (19). This effect increased after 2 years of intervention.

However, the current treatment of sirolimus is relatively single, with unclear treatment durations and certain adverse effects, such as oral ulcers, acne-like skin changes, hyperlipidemia, and menstrual disorders (14). Multimodal studies on mTOR pathway-related genes, including TSC1 and TSC2, may also provide valuable insights for future translational therapies (20). Other possible side effects include edema, fever, infections (pulmonary or other sites), gastrointestinal reactions, and slow wound healing. As surgeons, we believe that when sirolimus resistance or intolerance occurs, combining surgical treatment may be a new option. For giant AML, as in this case, preoperative use of sirolimus may effectively control or reduce tumor volume, facilitating successful NSS and achieving a “neoadjuvant chemoradiotherapy” effect. This direction warrants further clinical observation and research, especially for TSC-AML with bilateral multiple lesions rather than malignancy, where surgery may not be the first choice (7). Of course, when the diagnosis of LAM or TSC is unclear, the pros and cons of long-term sirolimus treatment need to be carefully weighed. Giant AML still carries the risk of rupture and hemorrhage during drug treatment, and related research or reports are rare.

In reviewing this case, we also had some questions. Based on preoperative imaging, the patient was found to have a uterine fibroid. Due to a lack of further supporting evidence, we were unable to determine whether its presence was related to LAM. However, considering that both LAM and uterine fibroids are hormonally responsive conditions, we advised the patient to closely monitor the progression of the fibroid, though no specific intervention was required at this time. Additionally, the ascites observed on imaging was not deemed significant for differential diagnosis and was likely incidental. Nonetheless, the potential for postoperative complications such as lymphatic edema warrants careful postoperative surveillance (21).

Although preoperative selective arterial embolization was effective, the degree of intraoperative bleeding exceeded our expectations. We suspect that microvenous and surrounding collateral vessels may have been the main source of bleeding. This highlights the higher demands placed on surgeons for complex AML surgeries. Preoperative careful review of imaging, understanding the tumor base location, distribution of renal arteries and veins, and planning the dissection range in advance are crucial for laparoscopic surgery of complex renal AML (22). The minimally invasive merits of robot-assisted partial nephrectomy or tumor enucleation must be balanced against the inherent challenges of giant AMLs with significant rupture and hemorrhage potential, where substantial intraoperative blood loss may mandate open surgical conversion. Therefore, thorough preoperative preparation is essential, including but not limited to: patient communication, blood preparation, and intraoperative assistance. In the future, there is still significant room for exploration and value in surgical techniques and combined drug-surgical treatments for complex AML.

The critical clinical takeaways from this case include two main points. First, a comprehensive understanding of the patient's auxiliary examinations is essential to accurately identify the underlying cause and support the most appropriate clinical decision-making. Second, staying current with advances in clinical research is crucial—particularly for surgeons—as the timing of surgical intervention can significantly influence outcomes. In the future, surgical approaches may increasingly be integrated with more diverse therapeutic modalities.

For complex and rare diseases like LAM with AML, only through careful and comprehensive diagnosis using clinical, imaging, and laboratory methods can the rate of misdiagnosis and missed diagnosis be effectively reduced. With the continuous exploration and clinical application of new drugs like mTOR inhibitors, complementing the advantages of medical and surgical treatments, and establishing systematic, rational, and regular management, we believe that more clinical patients will benefit in the future.

## 4 Patient perspective

Navigating the diagnosis and treatment of a rare and unfamiliar condition was initially overwhelming. However, the patient's strong educational background and high level of engagement allowed for open and thoughtful communication throughout her care. She actively sought expert opinions from multiple specialties, including urology, pulmonology, and pathology, which helped her feel informed and empowered in decision-making. After the first stage of surgery (Table 4), she continued her journey by consulting the LAM specialist team at Peking Union Medical College Hospital, where she underwent VEGF-D testing—an important step that provided clarity and direction for future management.

**Table 4:** Timeline of clinical course.

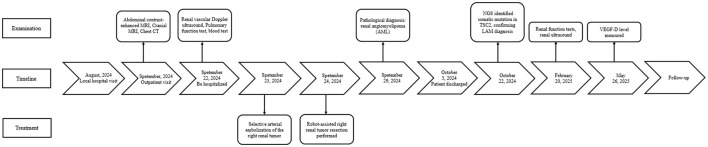

This table outlines the major examinations and treatments in chronological order, helping readers better understand the overall clinical course.

Throughout the process, the patient remained optimistic. She tolerated treatment well, experienced no significant decline in her quality of life, and expressed relief and hope after learning that no immediate medication was necessary. She described feeling grateful for the coordinated care she received and looks forward to the future with renewed confidence.

## Data Availability

The original contributions presented in the study are included in the article/supplementary material, further inquiries can be directed to the corresponding authors.
